# Gene expression of the zinc transporter ZIP14 (SLC39a14) is affected by weight loss and metabolic status and associates with PPARγ in human adipose tissue and 3T3-L1 pre-adipocytes

**DOI:** 10.1186/s40608-015-0076-y

**Published:** 2015-11-24

**Authors:** Trine Maxel, Kamille Smidt, Agnete Larsen, Marianne Bennetzen, Karina Cullberg, Karen Fjeldborg, Sten Lund, Steen B. Pedersen, Jørgen Rungby

**Affiliations:** Department of Biomedicine, Faculty of Health, Aarhus University, Aarhus, Denmark; Department of Clinical Medicine, Faculty of Health, Aarhus University, Aarhus, Denmark; Department of Endocrinology (MEA), Aarhus University Hospital, Aarhus, Denmark; Department of Medicine, Center for Diabetes Research, Gentofte University Hospital, Hellerup, Denmark

**Keywords:** SLC39a14, ZIP14, Adipose tissue, Obesity, Adipogenesis

## Abstract

**Background:**

The expansion and function of adipose tissue are important during the development of insulin resistance and inflammation in obesity. Zinc dyshomeostasis is common in obese individuals. In the liver, zinc influx transporter ZIP14, affects proliferation and glucose metabolism but the role of ZIP14 in adipose tissue is still unknown. This study investigates *ZIP14* gene expression in human adipose tissue before and after weight loss as well as the regulation of *ZIP14* during early adipogenesis.

**Methods:**

Fourteen obese individuals were investigated before and after a 10 week weight loss intervention and compared to 14 non-obese controls. Gene expressions of *ZIP14* and *peroxisome proliferator-activated receptor γ* (*PPARγ*) were measured in subcutaneous adipose tissue and correlated with metabolic and inflammatory markers. Further, we investigated gene expression of *ZIP14* and *PPARγ* during early adipogenesis of 3T3-L1 pre-adipocytes, together with an in silico analysis of PPARγ binding motifs in the promoter sequence of ZIP14.

**Results:**

*ZIP14* was down-regulated in obese individuals compared to non-obese controls (*p* = 0.0007) and was up-regulated after weight loss (*p* = 0.0005). Several metabolic markers of clinical importance, including body mass index, triglyceride, and insulin resistance, were inversely correlated with *ZIP14*. During early adipogensis an up-regulation of ZIP14 gene expression was found. *PPARγ* gene expression was positively correlated with the *ZIP14* gene expression in both adipose tissue and during adipogenesis. However, *in silico* analysis revealed that the ZIP14 promoter does not contain PPARγ-binding motifs.

**Conclusions:**

We hypothesize that *ZIP14*-mediated zinc influx might directly influence PPARγ activity and that *ZIP14* may regulate expansion and function of adipose tissue and serve as a potential biomarker for metabolic stress.

**Electronic supplementary material:**

The online version of this article (doi:10.1186/s40608-015-0076-y) contains supplementary material, which is available to authorized users.

## Background

White adipose tissue is an active endocrine organ that secretes a vast number of adipokines including inflammatory cytokines and adipose tissue is considered to be the main contributor to the low-grade inflammation and insulin resistance in obesity [1].

In addition to obesity-induced inflammation and insulin resistance, obese individuals have an altered zinc homeostasis and reduced levels of circulating zinc [2]. Serum zinc levels inversely correlate to insulin resistance [3]. In obese individuals, low dietary zinc intake relates to a disturbed lipid profile, a pronounced inflammatory response, and increased insulin production [4]. Intracellular zinc homeostasis is also important for adipose tissue function. In rat adipocytes, intracellular zinc has been shown to stimulate the insulin signaling pathway, leading to increased glucose uptake, enhanced lipogenesis and reduced production of free fatty acids [5].

Two families of zinc transporters, SLC39A (ZIPs) and SLC30A (ZnTs), control intracellular zinc influx and efflux from intracellular compartments as well as between the extracellular and intracellular environment [6]. Currently, 14 influx transporters (ZIP1–14) and ten efflux transporters (ZnT1–10) have been identified [7, 8]. The expression of the SLC39A family members *ZIP1*–*8* and their zinc-efflux controlling counterparts *ZnT1*–*8* have been examined in adipose tissue finding evidence that intracellular zinc homeostasis is altered in obese individuals compared with normal weight controls [9].

ZIP14 is a zinc- and iron-importing protein highly expressed in liver and pancreatic tissue, but it is also expressed in adipose tissue [10–12]. ZIP14 seems to have an impact on glucose homeostasis and adipose tissue, as ZIP14 knock-out mice shows an altered glucose homeostasis with hypoglycemia, increased insulin and liver glucose levels as well as increased adipose tissue [13]. Zinc is essential for cell differentiation and proliferation and ZIP14 has recently been shown to play an important role in the differentiation of hepatocytes and chondrocytes [13–18]. Moreover, ZIP14 displays a strong up-regulation during the early differentiation of murine 3T3-L1 pre-adipocytes into mature adipocytes, indicating a role for this transporter in adipogenesis [12].

In adipose tissue, the cell formation by adipogenesis, the formation of new adipocytes from precursor cells, is believed to constitute a healthy form of tissue expansion in contrast to hypertrophy of existing adipocytes [19]. The process is controlled by peroxisome proliferator-activated receptor γ (PPARγ) through its functions as a transcription factor, by which it regulates several genes involved in lipid and glucose metabolism [20, 21]. Pharmacological targeting of PPARγ has been attempted with the use of thiazolidinediones. Thiazolidinediones stimulate PPARγ and is considered to promote the formation of smaller adipocytes, known to be more sensitive to insulin and less lipolytically active than large hypertrophic adipocytes [22, 23].

The aim of this study was to examine the expression of ZIP14 *in vivo* in human adipose tissue relating the expression of this gene to body weight and clinical relevant markers of glucose and lipid metabolism, insulin resistance and PPARγ, thereby investigating the potential role for ZIP14 as a biomarker in metabolic diseases. We looked at obese individuals before and after weight loss as well as obese vs. non-obese individuals. Secondly, we sought to confirm the up-regulation of *ZIP14* in 3T3-L1 pre-adipocytes during early differentiation and investigate the correlation with differentiation markers, including PPARγ.

## Methods

### Participants and intervention in the weight loss study

The weight loss intervention study was performed at the Research Laboratory of Aarhus University Hospital. The study design and the assessment of clinical parameters (anthropometrics, insulin resistance, and lipid profile) have previously been described in detail [24]. The study included male and female participants, as well as age- and sex-matched non-obese controls. All participants were of European descent.

In summary, the obese participants had a body mass index (BMI) higher than 30 kg/m^2^. The non-obese controls had a BMI of 21–27 kg/m^2^. Subjects taking medication known to affect adipose tissue metabolism and/or with diagnosed type 2 diabetes were excluded from this study. In the present study, we examined 14 healthy, but obese, subjects (7 women and 7 men; age, 39 ± 7 years, BMI, 37 ± 3 kg/m^2^) and 14 non-obese controls (7 women and 7 men; age, 39 ± 9 years, BMI, 23 ± 2 kg/m^2^). The age range was 22–49 years.

Clinical investigations were performed at baseline and after 10 weeks (8 weeks of diet-induced weight loss followed by a 2-week weight-stabilizing period, during which the participants followed a normal diet adjusted to their new weight). During the weight loss period, participants followed a liquid, very low-calorie diet, which consisted of 600 calories together with 200 g of fruit and vegetables daily. Prior to clinical investigations, participants fasted overnight and refrained from excessive physical exercise and alcohol consumption for a 24 h period. The non-obese controls did not participate in the weight loss intervention but were examined once. This study was approved by the Central Denmark Region Committees on Biomedical Research Ethics and the Danish Data Protection Agency.

Anthropometrics (BMI, waist circumference, and hip circumference) and body fat percentage (by bioimpedance) were measured. Subcutaneous adipose tissue samples used for real-time PCR were obtained by liposuction from the abdomen, just below the umbilicus. Fasting blood samples were drawn from all participants and analyzed by standard procedures at the Department of Clinical Biochemistry, Aarhus University Hospital (glucose and lipids), except for serum insulin concentrations, which were measured by an ELISA (DAKO K6219, Electra Box Diagnostics Aps, Rødovre, Denmark) according to the manufacturer’s protocol. To evaluate insulin resistance, the homeostasis model assessment (HOMA) index was used (fasting serum insulin concentration (μU/L) × fasting plasma glucose concentration (mmol/L)/22.5) [25, 26]. The procedures were described previously [24].

Details on how the measurements of interleukin (IL)-6, leptin, and adiponectin in blood and *tumor necrosis factor* (*TNF*)*-α*, *IL-10*, as well as *leptin* gene expressions in adipose tissue are found and described in earlier studies [27, 28].

### Measurement of zinc and iron in blood samples

Serum zinc (Zn), serum iron (Fe), serum ferritin, and plasma transferrin were measured at the Department of Clinical Biochemistry, Randers Hospital, Denmark using a cobas system (COBAS^R^, using the e601 module for ferritin, otherwise the c501 module).

### 3T3-L1 cell culture and differentiation

Murine 3T3-L1 pre-adipocytes obtained from the American Type Culture Collection (Manassas, VA, USA) were cultured in 5 % CO_2_ in Dulbecco’s modified Eagle’s medium (DMEM) (Sigma-Aldrich, Denmark) containing 25 mM glucose, 10 % calf serum (PAA Cell Culture Company, Germany) and 1 % penicillin/streptomycin. Cells were cultured until 100 % confluent in 24-well plates. Two days after reaching confluency, cells were induced to differentiate into mature adipocytes by culture in DMEM containing 10 % fetal calf serum (Gibco, Denmark), 1 % penicillin/streptomycin, 1 μg/ml insulin (Sigma-Aldrich), 0.5 mM 3-isobutyl-1-methylxanthine (Sigma-Aldrich), and 1 μM dexamethasone (Sigma-Aldrich) [29]. Samples (*n* = 6 per group) were collected at 0, 1, 3, 6, 12, 24, and 48 h after differentiation began, which corresponds to the early differentiation phase, during which cells complete post-confluent mitosis to unwind DNA, thereby allowing transcription factors to regulate genes and resulting in the formation of cells resembling mature adipocytes [30]. Additional cells were grown until complete differentiation at day 8 to confirm correct differentiation (*n* = 6). The morphology of 3T3-L1 cells was examined throughout the entire differentiation process (i.e., until day 8) using an Olympus CKX41 camera and Olympus DP-Soft CVI version 3.2 software. Differentiation was further confirmed by measuring gene expression of the differentiation markers *PPARγ* and *fatty acid-binding protein 4 (A-FABP*) [30, 31].

### RNA extraction and real-time PCR

RNA was purified using TRIzol (Invitrogen, Denmark) according to the manufacturer’s instructions and was reverse-transcribed into cDNA using the ImProm-IITM Reverse Transcription System (Promega, Denmark). Two samples, one of 3T3-L1 cells and one of adipose tissue, were discarded due to DNA contamination.

The primers used are listed in Additional file 1 (TAC) [31, 32]. Quantitative real-time PCR was performed in duplicate with IQ SYBR Green Supermix (Bio-Rad, Denmark) in a MyiQ Single-Color Real-Time PCR Detection System (Bio-Rad). The results were analyzed with iQ™5 Optical System Software, version 2.0. Starting quantities were calculated from a standard curve. Values were normalized to the geomean of the housekeeping genes. The GeNorm method was used to confirm the stability of the housekeeping gene*s* [33]. *Low density lipoprotein receptor-related protein* 10 (LRP10) was specifically used as a housekeeping gene in the human study because of its known stability in human adipose tissue, which was confirmed in this experiment as well [32].

### In silico analysis of PPARγ-binding motifs in the promoter sequence of *ZIP14*

In silico analysis to identify PPARγ-binding motifs (PPARγ, PPARγ:RXRα, and (PPARγ:RXRα, PPARγ)) in the promoter sequence of human and mouse *ZIP14* was performed using MotifMap software (http://motifmap.ics.uci.edu/).

### Statistical analysis

#### Human study

PCR data from adipose tissue are provided as the mean starting quantity of (gene of interest/*LRP10)* ± standard error of the mean (SEM). Data were considered normally distributed through statistical analysis using the GraphPad Prism 5 program and a paired t-test was used to detect statistical difference between obese participants before and after weight loss. An unpaired t-test was used to detect statistical difference between the non-obese controls and the obese participants before weight loss. The standard Pearson’s correlation was calculated to detect possible correlations between *ZIP14* gene expression*,* and metabolic and inflammatory markers (adipokines) in obese individuals before weight loss and non-obese controls. Likewise, possible correlations were tested between gene expression of *ZIP14* and zinc or iron concentrations (transferrin, ferritin, total zinc, and total iron). A two-way analysis of variance (ANOVA) was used to evaluate any effect of gender on gene expression of *ZIP14*, *PPARγ1*, or *PPARγ2* comparing obese before weight loss and non-obese individuals.

#### In vitro study

PCR data from 3T3-L1 cells are provided as the mean starting quantity of (gene of interest/three housekeeping genes) ± SEM. Data were considered normally distributed through statistical analysis. A one-way ANOVA was performed, followed by the Newman-Keuls test if the result was significant, to detect statistically significant differences among the time points. The standard Pearson’s correlation was calculated to detect correlations between gene expression of the differentiation markers (*PPARγ* and *A-FABP*) and that of *ZIP14* during differentiation.

For all statistical analyses, a *p-*value ≤ 0.05 was considered significant and the statistical package GraphPad Prism 5 was used for calculations.

## Results

### Part I: Human study. Expression of *ZIP14* and *PPARγ* in adipose tissue of obese individuals before and after weight loss and comparisons with non-obese individuals: correlations with anthropometric, metabolic, and inflammatory markers

At baseline, all obese participants had a BMI higher than 30 kg/m^2^ (37 ± 3 kg/m^2^), while non-obese controls had a BMI of 21–27 kg/m^2^ (23 ± 2 kg/m^2^). Comparisons of baseline data showed the expected differences in waist/hip ratio, body fat percentage, and glucose and lipid profiles between obese individuals and non-obese controls (Table 1), see also [24].

**Table 1:** Metabolic data of obese and non-obese individuals

	Obese individuals before weight loss	Non-obese controls	Obese individuals after weight loss
BMI (kg/m^2^)	37 (±3)	23 (±2)	33 (±3)
		***	***
Waist/hip ratio	1.0 (±0.09)	0.8 (±0.08)	0.9 (±0.08)
		***	***
Body fat percentage	42 (±6)	23 (±7)	38 (±6)
		***	***
Cholesterol (mmol/L)	5.4 (±1.1)	4.9 (±0.8)	4.9 (±0.8)
			***
HDL (mmol/L)	1.1 (±0.2)	1.5 (±0.3)	1.1 (±0.2)
		***	
LDL (mmol/L)	3.4 (±0.8)	2.9 (±0.8)	2.9 (±0.6)
			***
Triglyceride (mmol/L)	2.1 (±0.9)	1.1 (±0.4)	1.9 (±0.8)
		***	
Insulin (pmol/L)	92 (±28)	49 (±15)	67 (±32)
		***	***
Glucose (mmol/L)	5.8 (±0.5)	5.3 (±0.4)	5.6 (±0.2)
		**	
HOMA index	4.0 (±1.3)	1.9 (±0.6)	2.8 (±1.3)
		***	***

Anthropometric data, lipid profiles, and glucose homeostasis in blood from obese individuals before and after weight loss and from non-obese controls. Data are presented as mean ± standard deviation (*n* **=** 14 per group). **, *P* < 0.01; ***, *P* < 0.001 compared with obese individuals before weight loss. BMI, body mass index; HDL, high-density lipoprotein; HOMA, homeostasis model assessment; LDL, low-density lipoprotein

#### Anthropometric and metabolic data improved after weight loss

The obese individuals obtained a weight loss of 12.5 ± 3.4 kg on average and a significant reduction in BMI from 37 ± 3 kg/m^2^ to 33 ± 3 kg/m^2^. The weight loss was reflected by decreased body fat percentage (*p* < 0.0001) as well as decreased waist/hip ratio (*p* < 0.0001).

Cholesterol (*p* = 0.0005) and low-density lipoprotein (LDL; *p* = 0.0005) concentrations significantly decreased after weight loss. The triglyceride concentration was only significantly reduced in women (*p* = 0.0213), whereas there was no change in the high-density lipoprotein (HDL) concentration.

The fasting insulin concentration (*p* = 0.0002) and insulin resistance (*p* = 0.0002) as judged by the HOMA index were significantly reduced after weight loss, although the glucose concentration did not change. Anthropometric and metabolic data of the participants are shown in Table 1, see also [24].

#### *ZIP14* gene expression is significantly lower in obese individuals than in non-obese individuals, and increases markedly following weight loss

**Fig. 1:**
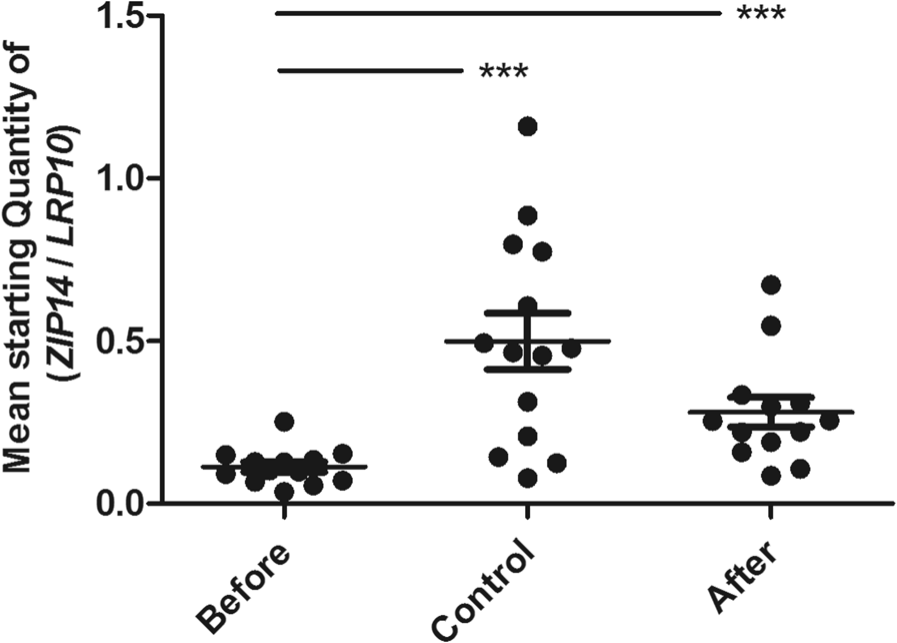
Gene expression of *ZIP14* in adipose tissue. Gene expression of *ZIP14* in adipose tissue from obese individuals before (Before) and after (After) weight loss and from non-obese controls (Control). Data are presented as the mean starting quantity of (*ZIP14*/*LRP10*) ± standard error of the mean (*n* = 13–14 per group). ***, *P* < 0.001

At baseline, *ZIP14* gene expression was significantly lower in obese individuals than in non-obese controls (*p* = 0.0007). After weight loss, *ZIP14* gene expression significantly increased (*p* = 0.0005) (Fig. 1). Two-way ANOVA showed no independent influence of gender on *ZIP14* gene expression (data not shown).

#### The expression patterns of *PPARγ1* and *ZIP14* are similar

**Fig. 2:**
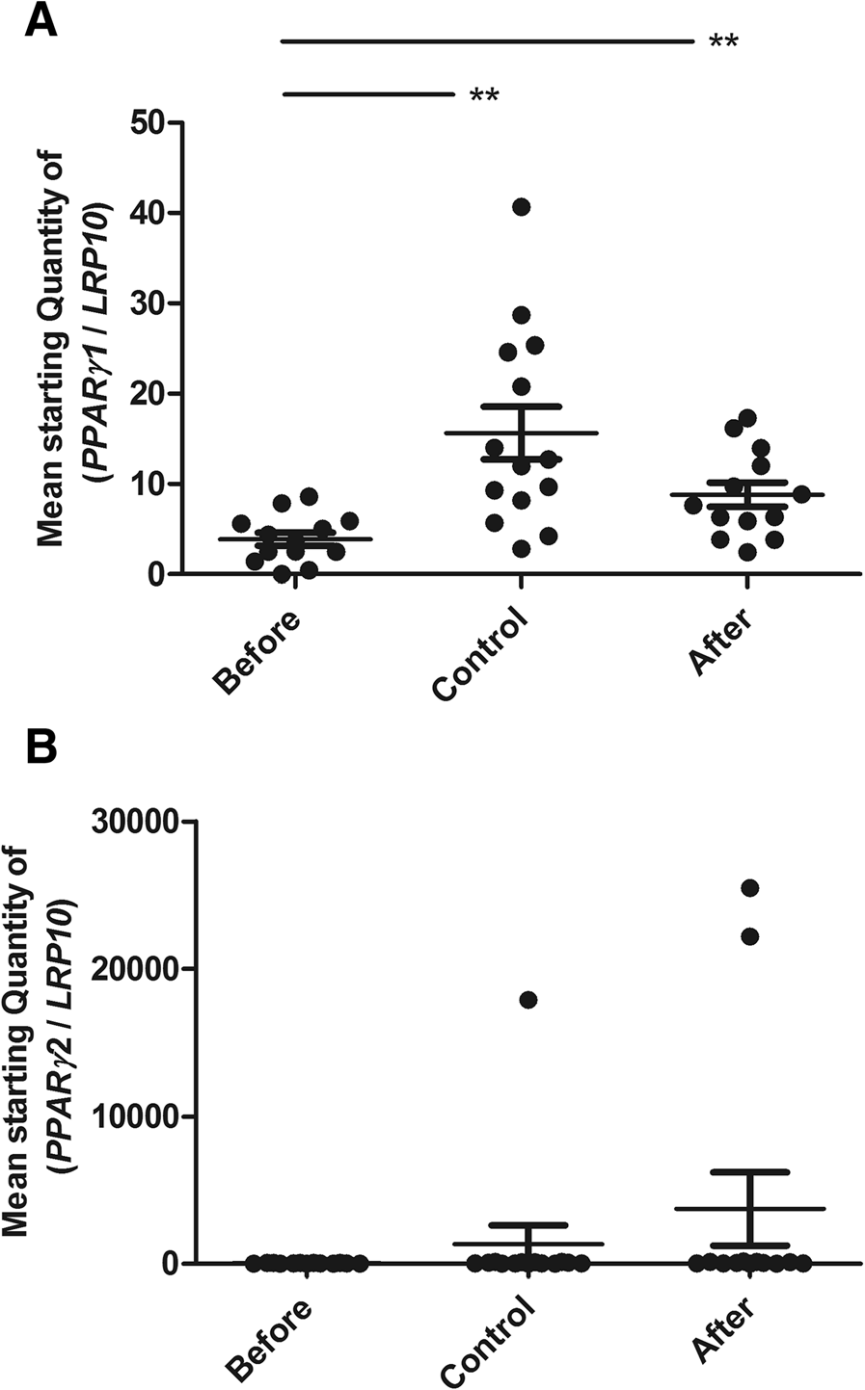
Gene expression of *PPARγ1* and *PPARγ2* in adipose tissue. Gene expression of *PPARγ1* (**a**) and *PPARγ2* (**b**) in adipose tissue from obese individuals before (Before) and after (After) weight loss and from non-obese controls (Control). Data are presented as the mean starting quantity of (gene of interest/*LRP10*) ± standard error of the mean (*n* = 13–14 per group). **, *P* < 0.01

The two main isoforms of *PPARγ* mRNA, namely, *PPARγ1* and *PPARγ2*, were separately investigated. *PPARγ1* gene expression was significantly lower in obese individuals than in non-obese controls (*p* = 0.0016). After weight loss, *PPARγ1* gene expression significantly increased (*p* = 0.0018) (Fig. 2a). Gene expression of *PPARγ2* did not significantly differ (Fig. 2b).

Two-way ANOVA showed no effect of gender on the gene expression of *PPARγ1* or *PPARγ2*.

#### Total blood levels of zinc and iron are unaffected by body weight

Levels of zinc and markers of iron homeostasis in blood are significantly lower in women than in men (see also Table 2) [34, 35]; therefore, we examined the two genders separately. At baseline, Zn, Fe, and transferrin concentrations did not differ between obese individuals and non-obese controls (neither before nor after weight loss), and weight loss did not significantly alter these concentrations in the obese individuals. The concentration of ferritin was significantly higher in obese men compared to non-obese men both before and after weight loss (*p* = 0.0243 and *p* = 0.0332, respectively), but no significant effect of a weight loss was found. The concentrations of Zn, Fe, transferrin, and ferritin are shown in Table 2.

**Table 2:** Zinc and iron homeostatic markers in blood from obese and non-obese individuals

	Obese individuals before weight loss		Non-obese controls		Obese individuals after weight loss	
	Women	Men	Women	Men	Women	Men
Zn (μmol/L)	13.3 (±1.7)	14.3 (±1.3)	11.8 (±0.7)***	13.3 (±0.1)	12.8 (±1.7)	14.5 (±1.4)
Fe (μmol/L)	17.4 (±5.5)	21.9 ( ±6.9)	15.8 (±5.2)**	22.7 (±5.1)	13.1 (±5.6)	18.8 (±5.2)
Ferritin (μg/L)	61 (±40)****	247 (±105)*	49 (±43)	120 (±78)	69 (±51)***	243 (±111)*
Transferrin (μmol/L)	36.0 (±3.6)	33.7 (±3.9)	34.4 (±6.8)	34.2 (±4.7)	33.6 (±3.3)	32.2 (±3.8)

Total Zn, Fe, ferritin, and transferrin concentrations in blood from obese individuals (before and after weight loss) and from non-obese controls. Data are presented as mean ± standard deviation. *P < 0.05, obese individuals vs. non-obese controls of the same gender. **P < 0.05, ***P < 0.01, ****P < 0.001, when comparing women vs men within the same weight group. No significant differences were found when comparing obese individuals before and after weight loss

#### Gene expression of *ZIP14* correlates with clinical signs of metabolic dysfunction and *PPARγ* gene expression

There was a significant positive correlation between gene expression of *ZIP14* and that of *PPARγ1* (*p* < 0.0001) and *PPARγ2* (*p* = 0.0487). In terms of clinical parameters, *ZIP14* gene expression was significantly inversely correlated with all markers of obesity, namely, BMI (*p* = 0.0012), waist/hip ratio (*p* = 0.0181), and body fat percentage (*p* = 0.0009). Moreover, *ZIP14* gene expression was inversely correlated with HOMA index (*p* = 0.0009), blood glucose (*p* = 0.0324) and insulin (*p* = 0.0007). Further, there was a significant positive correlation between *ZIP14* gene expression and HDL concentration (*p* = 0.0015), whereas triglyceride concentration was significantly inversely correlated with *ZIP14* gene expression (*p* = 0.0041). There were no significant correlations between *ZIP14* gene expression and LDL or cholesterol concentrations. Pearson’s r-values and *p-*values are shown in Table 3.

**Table 3:** Correlations between *ZIP14* gene expression, *PPARγ* gene expression*,* and metabolic markers

	*ZIP14* gene expression
*PPARγ1* gene expression	r = 0.68, *p* < 0.0001
*PPARγ2* gene expression	r = 0.38, *p* = 0.0487
BMI	r = −0.59, *p* = 0.0012
Waist/hip ratio	r = −0.45, *p* = 0.0181
Body fat percentage	r = −0.60, *p* = 0.0009
Cholesterol	r = −0.25, *p* = 0.2112
HDL	r = 0.58, *p* = 0.0015
LDL	r = −0.26, *p* = 0.1925
Triglyceride	r = −0.53, *p* = 0.0041
Insulin	r = −0.61, *p* = 0.0007
Glucose	r = −0.41, *p* = 0.0324
HOMA index	r = −0.60, *p* = 0.0009

Correlations between *ZIP14* and *PPARγ* gene expression levels, anthropometric data, lipid profiles, and glucose homeostasis from obese individuals before weight loss and from non-obese controls (*n* = 27). Pearson’s r-values and *p*-values are shown. BMI, body mass index; HDL, high-density lipoprotein; HOMA, homeostasis model assessment; LDL, low-density lipoprotein; PPAR*, peroxisome proliferator-activated receptor*

There were no significant correlations between concentrations of Zn, Fe, transferrin, and ferritin with *ZIP14* gene expression (data not shown). All correlations included obese individuals before weight loss and non-obese controls.

#### *ZIP14* gene expression in adipose tissue correlates with several adipokines in adipose tissue and blood

As previously described [27, 28], in adipose tissue *TNF-α, IL-10,* as well as *leptin* gene expression was significantly higher in obese individuals than in non-obese controls. IL-6 levels in blood however showed no significant difference between obese and non-obese individuals. Blood concentrations of leptin and adiponectin were significantly higher and lower, respectively, in obese individuals than in non-obese controls.

There was an inverse correlation between gene expression of *ZIP14* and that of *TNF-α*, *leptin*, and *IL-10* in adipose tissue (*p* = 0.0241, *p* < 0.0001, and *p* = 0.0068, respectively) and the level of leptin in blood (*p* = 0.0040) (Table 4). *ZIP14* gene expression showed no significant correlations with IL-6 or adiponectin in blood. All correlations included obese individuals before weight loss and non-obese controls.

**Table 4:** Correlations between *ZIP14* gene expression and the gene expression and blood levels of adipokines

	*ZIP14* gene expression
*TNF-α* gene expression	r = −0.44, *p* = 0.0241
*Leptin* gene expression	r = −0.70, *p* < 0.0001
*IL-10* gene expression	r = −0.53, *p* = 0.0068
IL-6 (blood level)	r = −0.31, *p* = 0.1059
Leptin (blood level)	r = −0.54, *p* = 0.0040
Adiponectin (blood level)	r = −0.05, *p* = 0.8127

Correlations between *ZIP14* gene expression in adipose tissue and blood levels and gene expression in adipose tissue of adipokines. Obese individuals before weight loss and non-obese controls were included (*n* = 27). Pearson’s r-values and *p*-values are shown. IL, interleukin; TNF, tumor necrosis factor

### Part II: *In vitro* regulation of *ZIP14* during the early differentiation of 3T3-L1 cells

We investigated the gene expression of *ZIP14* in 3T3-L1 pre-adipocytes during the early differentiation phase (0, 1, 3, 6, 12, 24 and 48 h after the induction of differentiation).

#### Up-regulation of *A-FABP* and *PPARγ* gene expression confirms that 3T3-L1 cells differentiated into mature adipocytes

**Fig. 3:**
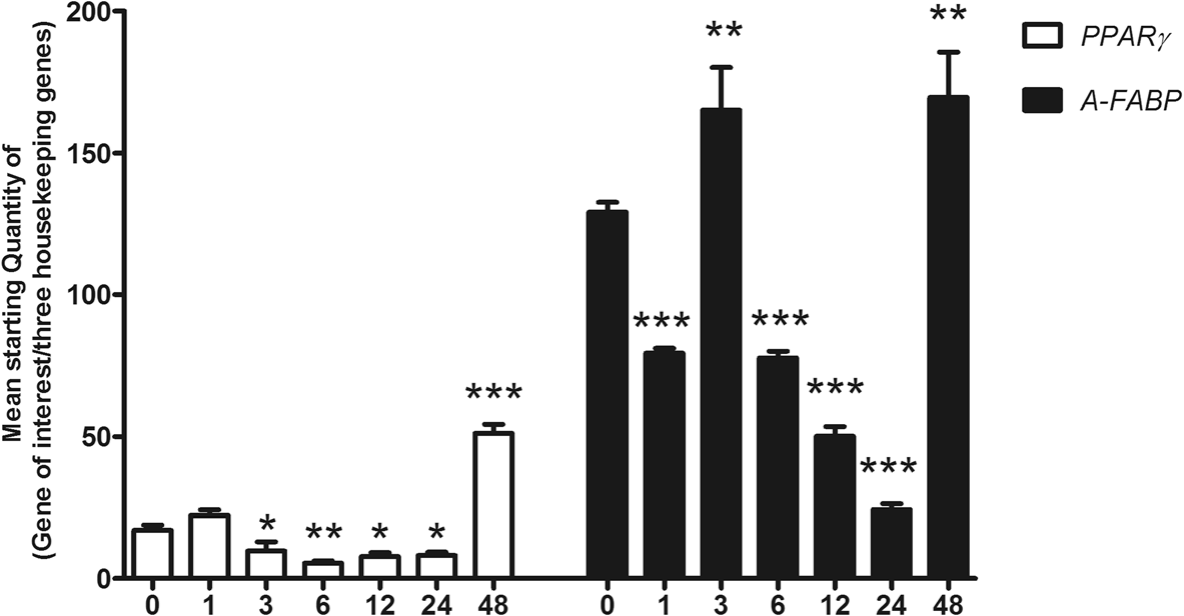
Gene expression of differentiation markers in 3T3-L1 cells during the early differentiation phase. Gene expression of the differentiation markers *peroxisome proliferator-activated receptor γ* (*PPARγ*) and *fatty acid-binding protein 4* (*A-FABP*). Results are expressed as the mean starting quantity of (gene of interest/three housekeeping genes) ± standard error of the mean. Data are shown during the early differentiation phase (0, 1, 3, 6, 12, 24 and 48 h after the induction of differentiation), (*n* = 5–6 per group). *, *P* < 0.05; **, *P* < 0.01; ***, *P* < 0.001. Significant differences are shown compared to undifferentiated cells (0 h)

*A-FABP* and *PPARγ* were chosen as adipocyte differentiation markers [30, 31]. Both genes were significantly up-regulated 48 h after differentiation began and confirmed differentiation (*p* < 0.01 compared with levels prior to differentiation (0 h)) (Fig. 3) [31]. Microscopy analysis of cells 8 days after initiating differentiation showed the transformation of 3T3-L1 cells into mature adipocytes compared with differentiation start (Additional file 2).

#### Gene expression of *ZIP14* is up-regulated during early differentiation and correlates with *PPARγ*

**Fig. 4:**
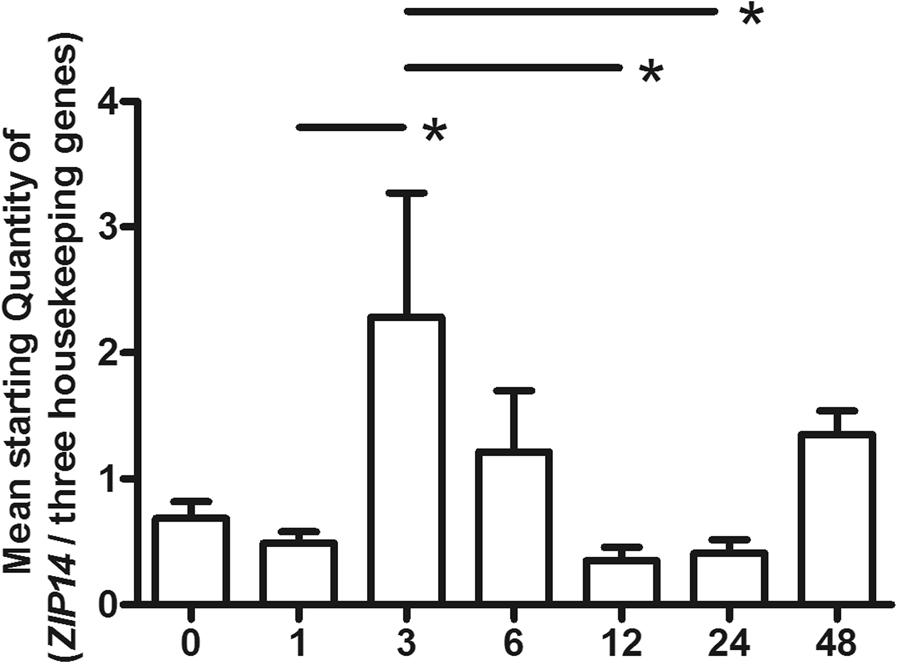
Gene expression of *ZIP14* in 3T3-L1 cells during the early differentiation phase. Gene expression of *ZIP14*. Results are expressed as the mean starting quantity of (*ZIP14*/three housekeeping genes) ± standard error of the mean. Data are shown during the early differentiation phase (0, 1, 3, 6, 12, 24 and 48 h after the induction of differentiation), (*n* = 5–6 per group). *, *P* < 0.05

Gene expression of *ZIP14* peaked 3 h after initiating differentiation (Fig. 4). When correlating the gene expression of *ZIP14* with that of *PPARγ* at the time of the peak (3 h) showed a significant positive correlation (*p* = 0.0143). No correlation between *ZIP14* and *A-FABP* was seen at this time point. No significant correlations were found between *ZIP14* gene expression and the two differentiations markers before the peak (0–1 h), but after the peak both differentiation markers, *PPARγ* and *A–FABP*, showed significant positive correlations with *ZIP14* (6–48 h) (*p* = 0.0406 and *p* = 0.0484, respectively (Table 5).

**Table 5:** Correlations between *ZIP14* gene expression and the gene expression of differentiation markers

*PPARγ* gene expression	*ZIP14* gene expression
0-1 h	r = 0.44, *p* = 0.1481
3 h	r = 0.90, *p* = 0.0143
6-48 h	r = 0.43, *p* = 0.0406
*A-FABP* gene expression	*ZIP14* gene expression
0-1 h	r = 0.42, *p* = 0.1692
3 h	r = 0.38, *p* = 0.1205
6-48 h	r = 0.42, *p* = 0.0484

Correlations between *ZIP14* gene expression with *PPARγ* and *A-FABP* gene expression during early differentiation of 3T3-L1 cells. Correlations are shown before, during, and after the peak in *ZIP14* gene expression, corresponding to 0–1 h, 3 h, and 6–48 h after differentiation start. Pearson’s r-values and *p*-values are shown. A-FABP, fatty acid-binding protein 4; PPAR*, peroxisome proliferator-activated receptor*

### Part III: In silico analysis of *PPARγ*-binding motifs in the promoter sequence of *ZIP14*

To investigate the correlation between the gene expression patterns of *ZIP14* and *PPARγ* in both human adipose tissue and during differentiation, the promoter sequence of *ZIP14* was analyzed. MotifMap software did not find evidence of the presence of any PPARγ-binding motifs (PPARγ, PPARγ:RXRα, and (PPARγ:RXRα, PPARγ)) in the promoter sequence of human or mouse *ZIP14*.

## Discussion

The present study supports a role for *ZIP14*, a zinc and iron transporter, in adipogenesis and adipose tissue homeostasis. *ZIP14* gene expression was significantly lower in obese individuals than in non-obese controls. After weight loss, *ZIP14* gene expression significantly increased, indicating that regulation of *ZIP14* is related to obesity and that this process is reversible. Our findings indicate that gene expression of *ZIP14* is associated with that of *PPARγ*, which was shown in both the human adipose tissue as well as in the 3T3-L1 pre-adipocytes during differentiation *in vitro*. Furthermore, we linked the expression of *ZIP14* with several clinical markers of metabolism, including BMI, lipid profile, glucose homeostasis, and levels of inflammatory markers. Our results support a link between altered zinc homeostasis and metabolic disease, indicating that ZIP14 has a specific role in adipose tissue function and is a potential biomarker of metabolic stress in adipose tissue.

The down-regulation of *ZIP14* found in obese individuals in this study supports the notion that intracellular zinc regulation is altered in adipose tissue from obese individuals. Similar findings were described by Smidt and co-workers who investigated several zinc transporters of both families (*ZnT1-8* and *ZIP1-8*) and found the majority of them to be down-regulated in obesity [9]. Both the present study and the work of Smidt and co-workers were performed on biopsies of fatty tissues in which a degree of macrophage infiltration must be expected, especially in obese individuals [1]. The expression levels of the macrophage markers CD68 and CD14 have previously been assessed in this study population and showed a higher degree of macrophage infiltration in the obese compared with the non-obese controls [28]. Altered intracellular zinc regulation in macrophages might therefore also affect the findings. An up-regulation of *ZIP14* has been shown in macrophages during an acute inflammatory response [36] indicating a potential difference among cell types, however data from the *in vitro* experiment in the 3T3-L1 cells supports a major role of adipocytes in the down-regulation of *ZIP14*.

Recent studies suggest a function for ZIP14 in cell proliferation and differentiation. At the cellular level, ZIP14 over-expression enhances the proliferation of hepatocytes and ZIP14 is up-regulated during liver regeneration in a murine model of partial hepatectomy, with a concomitant increase in the hepatic zinc content, which is not seen in ZIP14 knock-out mice [16]. Further evidence of a role for ZIP14 in cell proliferation and differentiation was found in investigations of bone formation in ZIP14 knock-out mice, in which shortening of long bones was described together with disturbed chondrocyte differentiation [18].

In the current study, *ZIP14* was up-regulated during the early differentiation of adipocytes, during which cells undergo post-confluent mitotic cell division and DNA unwinding, which allows transcriptions factors to regulate genes that induce further differentiation into mature adipocytes. This indicates a function for ZIP14 protein in adipogenesis, confirming the finding of Tominaga *et al.* that *ZIP14* expression peaks during the early differentiation of 3T3-L1 cells [12].

Because of its regulation of DNA synthesis and mitosis, zinc is essential for cell proliferation and differentiation [14, 37]. In 3T3-L1 cells, zinc influx transiently increases by 5-fold 24 h after the induction of differentiation. This influx of zinc correlates with cell proliferation and seems to be essential for entry into S-phase of the cell cycle [38]. It could therefore be speculated that zinc influx (by ZIP14) is increased in dividing cells to regulate adipogenesis, with elevated *ZIP14* gene expression 3 h after the induction of differentiation, which corresponds to translation of RNA into protein, and a subsequent peak in zinc ion transport at 24 h [38]. We speculate that the decrease in ZIP14 gene expression occurs through a negative feedback regulation involving the metal-response element-binding transcription factor 1(MTF-1). MTF-1 is known to be induced upon increased cytosolic zinc levels and has been shown to regulate mRNA expression of another SLC39A transporter, ZIP10 in a negative manner in hepatocytes [39, 40].

To our best knowledge, the association between *ZIP14* and *PPARγ* gene expression has not been previously investigated. In our experiments, *ZIP14* and *PPARγ* expression appeared to be linked in both murine 3T3-L1 adipocytes and human adipose tissue. PPARγ is the main regulator of adipogenesis, and mature adipocytes require sustained *PPARγ* expression to remain in a differentiated state [20]. PPARγ is a transcription factor that forms a heterodimer with retinoid X receptor-α, followed by binding to peroxisome proliferator response elements in the promoter regions of target genes involved in lipid metabolism and adipogenesis [21]. Structurally, it requires zinc to form two zinc finger-like motifs. These motifs are responsible for binding to promoter regions in target genes [41]. It could be speculated that PPARγ controls *ZIP14* through its function as a transcription factor however our *in silico* analysis, did not support the presence of any PPARγ-binding motifs in the promoter sequence of *ZIP14*. As *ZIP14* gene expression peaked before *PPARγ* during early differentiation, an indirect link between *ZIP14* and *PPARγ* could be hypothesized *namely* that the zinc imported by ZIP14 could affect *PPARγ* activity. On the other hand: both *PPARγ* and *ZIP14* gene expression have been shown to be up-regulated by pro-inflammatory cytokines [42, 43]. As adipocytes secrete a vast number of cytokines, including TNF-α and IL-6, these inflammatory cytokines could cause an increase in both *ZIP14* and *PPARγ* expression [44].

Two splice variants of PPARγ have been described in human tissue, namely, PPARγ1 and PPARγ2. *PPARγ1* mRNA is highly expressed in adipose tissue but is also expressed in other tissues, whereas *PPARγ2* is restricted to adipose tissue [21]. We investigated these isoforms separately in human adipose tissue because other studies have found weight-dependent differences in gene expression of *PPARγ1* and *PPARγ2* in human adipocytes [45]. Gene expression of *ZIP14* correlated with that of both isoforms, but its correlation with *PPARγ1* gene expression was strongest.

In the present study, subcutaneous abdominal adipose tissue was investigated*.* Although this tissue is considered to be healthier than visceral adipose tissue, it is still associated with metabolic risk factors such as increased BMI and blood pressure and adverse glucose and lipid homeostasis [46]. Notably, *ZIP14* gene expression in this tissue was significantly correlated with several metabolic markers including anthropometric data (BMI, body fat percentage, and waist/hip ratio), lipid metabolism (triglyceride and HDL concentrations), and glucose metabolism (glucose concentration, insulin concentration, and the HOMA index). This indicates that ZIP14 and its regulation are linked to the metabolic function of the body, with down-regulation in obesity. Based on the present data, we cannot confirm any causative relationship between ZIP14 and clinical parameters. We can however speculate that since consistent correlations were found by investigating this limited number of individuals, there is a connection between ZIP14 and lipid and glucose metabolism, indicating a role for ZIP14 as a biomarker of metabolic stress in adipose tissue.

In support of this, *ZIP14* down-regulation was correlated with the expression of several cytokines produced in unhealthy adipose tissue as part of the inflammatory response in obesity, i.e., *TNF-α* and *IL-10*. Paradoxically, other studies have shown that *ZIP14* gene expression is up-regulated in liver, muscle, and white adipose tissue when a low-grade inflammatory state is induced in mice by lipopolysaccharide stimulation, possibly through interaction with IL-6 [13, 47]. In the present study, *ZIP14* gene expression showed no significant correlation with the IL-6 levels in the blood. However, it should be noted that the IL-6 expression levels of the obese individuals did not significantly differ from the levels found in their non-obese counterparts. Our findings may reflect a difference in regulation of *ZIP14* following an LPS induced- acute inflammatory state with a strong IL-6 response vs. the chronic inflammation found in obesity. Tissue specific pattern of inflammation and macrophage presence might also play a role.

As expected, levels of the weight regulatory hormones adiponectin and leptin were lower and higher, respectively, in obese individuals than in non-obese controls [48]. The levels of leptin in blood and adipose tissue were inversely correlated with gene expression of *ZIP14*. It has been known for many years that levels of zinc and leptin are correlated. Several studies investigating healthy, obese, or obese type 2 diabetic individuals have shown an inverse correlation between plasma leptin and zinc levels [49–51]. Our finding of an inverse correlation between *ZIP14* gene expression and the level of leptin suggests a complex relationship between leptin and zinc homeostasis that involves intracellular zinc homeostasis in adipose tissue, not just total zinc levels in blood.

No correlation was found between *ZIP14* gene expression and the total zinc level in blood. Although several studies confirmed that the circulating zinc level is reduced in obese individuals, zinc levels appear to be highly variable, even in obese individuals [52–54]. In the current study, the zinc concentration did not significantly differ between obese individuals and non-obese controls. This confirms a report that the level of zinc in erythrocytes, but not in plasma, differs between obese individuals and non-obese controls [55]. The serum zinc level must be interpreted with caution and is not generally considered to be a reliable biomarker of zinc status, but is often the only available measurement [34].

## Conclusion

In summary, the gene expression of *ZIP14*, a zinc and iron importer, is reversibly down-regulated in adipose tissue in chronic obesity and is inversely linked to several clinical markers of lipid and glucose homeostasis. Furthermore, gene expression of *ZIP14* is linked with adipogenesis, as suggested by its correlation with gene expression of the adipogenic transcription factor *PPARγ*. No PPARγ-binding motifs were found in the promoter sequence of *ZIP14*; and we suggest that the connection between *ZIP14* and *PPARγ* could be linked to the zinc-importing functions of ZIP14 during adipogenesis although a contribution from inflammatory cytokines cannot be excluded. We speculate that ZIP14 could prove to be not only a new biomarker of metabolic stress but possibly a future medical target in the treatment of obesity, affecting intracellular zinc regulation and adipogenesis and thereby supporting a healthy adipose tissue expansion.

## Consent and permission

This study was approved by the Central Denmark Region Committees on Biomedical Research Ethics and the Danish Data Protection Agency. All participants gave their written consent before investigation.

## Additional files

Additional file 1:
**Primer sequences.** Forward and reverse primer sequences are shown together with the annealing temperature. h, human primer; m, murine primer. In human adipose tissue, expression of the zinc transporter *ZIP14* was investigated together with that of *peroxisome proliferator-activated receptor γ* isoform 1 (*PPARγ1*) and isoform 2 (*PPARγ2*). *Low-density lipoprotein receptor-related protein 10 (LRP10*) was used as a housekeeping gene. In 3T3-L1 cells, expression of the zinc transporter *ZIP14* was investigated together with that of the adipocytic differentiation markers *PPARγ* and *fatty-acid binding protein 4 (A-FABP). Cyclophilin A (Cyc-A), hypoxanthine guanine phosphoribosyl transferase (HPRT),* and *ubiquitin conjugase-7 (UBC-7)* were used as housekeeping genes. (PDF 58 kb) Additional file 2:
**Micrograph of undifferentiated (left side) and mature adipocytes (right side).** Micrograph (×20) of 3T3-L1 cells prior to differentiation (left) and mature 3T3-L1 adipocytes 8 days after differentiation began (right). (TIF 1332 kb)

## Abbreviations

A-FABP: Fatty acid binding protein 4
BMI: Body mass index
HDL: High-density lipoprotein
HOMA: Homeostasis model assessment
IL: Interleukin
LDL: Low-density lipoprotein
LRP10: Low density lipoprotein receptor-related protein 10
MTF-1: Metal-response element-binding transcription factor 1
PPARγ: Peroxisome proliferator-activated receptor gamma
TNF-α: Tumor necrotic factor-α

## References

[1] Bastard JP, Maachi M, Lagathu C, Kim MJ, Caron M, Vidal H (2006). Recent advances in the relationship between obesity, inflammation, and insulin resistance. Eur Cytokine Netw.

[2] Garcia OP, Long KZ, Rosado JL (2009). Impact of micronutrient deficiencies on obesity. Nutr Rev.

[3] Ortega RM, Rodriguez-Rodriguez E, Aparicio A, Jimenez AI, Lopez-Sobaler AM, Gonzalez-Rodriguez LG (2012). Poor zinc status is associated with increased risk of insulin resistance in Spanish children. Br J Nutr.

[4] Costarelli L, Muti E, Malavolta M, Cipriano C, Giacconi R, Tesei S (2010). Distinctive modulation of inflammatory and metabolic parameters in relation to zinc nutritional status in adult overweight/obese subjects. J Nutr Biochem.

[5] Yoshikawa Y, Ueda E, Kojima Y, Sakurai H (2004). The action mechanism of zinc(II) complexes with insulinomimetic activity in rat adipocytes. Life Sci.

[6] Liuzzi JP, Cousins RJ (2004). Mammalian zinc transporters. Annu Rev Nutr.

[7] Huang L, Tepaamorndech S (2013). The SLC30 family of zinc transporters - a review of current understanding of their biological and pathophysiological roles. Mol Aspects Med.

[8] Jeong J, Eide DJ (2013). The SLC39 family of zinc transporters. Mol Aspects Med.

[9] Smidt K, Pedersen SB, Brock B, Schmitz O, Fisker S, Bendix J (2007). Zinc-transporter genes in human visceral and subcutaneous adipocytes: lean versus obese. Mol Cell Endocrinol.

[10] Jenkitkasemwong S, Wang CY, Mackenzie B, Knutson MD (2012). Physiologic implications of metal-ion transport by ZIP14 and ZIP8. Biometals.

[11] Taylor KM, Morgan HE, Johnson A, Nicholson RI (2005). Structure-function analysis of a novel member of the LIV-1 subfamily of zinc transporters, ZIP14. FEBS Lett.

[12] Tominaga K, Kagata T, Johmura Y, Hishida T, Nishizuka M, Imagawa M (2005). SLC39A14, a LZT protein, is induced in adipogenesis and transports zinc. FEBS J.

[13] Beker AT, Chang SM, Guthrie GJ, Maki AB, Ryu MS, Karabiyik A (2012). Zinc transporter ZIP14 functions in hepatic zinc, iron and glucose homeostasis during the innate immune response (endotoxemia). PLoS One.

[14] Beyersmann D, Haase H (2001). Functions of zinc in signaling, proliferation and differentiation of mammalian cells. Biometals.

[15] Kiss Z, Crilly KS, Tomono M (1997). Bombesin and zinc enhance the synergistic mitogenic effects of insulin and phosphocholine by a MAP kinase-dependent mechanism in Swiss 3 T3 cells. FEBS Lett.

[16] Aydemir TB, Sitren HS, Cousins RJ (2012). The zinc transporter Zip14 influences c-Met phosphorylation and hepatocyte proliferation during liver regeneration in mice. Gastroenterology.

[17] Liuzzi JP, Aydemir F, Nam H, Knutson MD, Cousins RJ (2006). Zip14 (Slc39a14) mediates non-transferrin-bound iron uptake into cells. Proc Natl Acad Sci U S A.

[18] Hojyo S, Fukada T, Shimoda S, Ohashi W, Bin BH, Koseki H (2011). The zinc transporter SLC39A14/ZIP14 controls G-protein coupled receptor-mediated signaling required for systemic growth. PLoS One.

[19] Sun K, Kusminski CM, Scherer PE (2011). Adipose tissue remodeling and obesity. J Clin Invest.

[20] Rosen ED, MacDougald OA (2006). Adipocyte differentiation from the inside out. Nat Rev Mol Cell Biol.

[21] Zieleniak A, Wojcik M, Wozniak LA (2008). Structure and physiological functions of the human peroxisome proliferator-activated receptor gamma. Arch Immunol Ther Exp (Warsz ).

[22] Yang X, Smith U (2007). Adipose tissue distribution and risk of metabolic disease: does thiazolidinedione-induced adipose tissue redistribution provide a clue to the answer?. Diabetologia.

[23] Tchernof A, Despres JP (2013). Pathophysiology of human visceral obesity: an update. Physiol Rev.

[24] Bennetzen MF, Wellner N, Ahmed SS, Ahmed SM, Diep TA, Hansen HS (2011). Investigations of the human endocannabinoid system in two subcutaneous adipose tissue depots in lean subjects and in obese subjects before and after weight loss. Int J Obes (Lond).

[25] Matthews DR, Hosker JP, Rudenski AS, Naylor BA, Treacher DF, Turner RC (1985). Homeostasis model assessment: insulin resistance and beta-cell function from fasting plasma glucose and insulin concentrations in man. Diabetologia.

[26] Volund A (1993). Conversion of insulin units to SI units. Am J Clin Nutr.

[27] Fjeldborg K, Christiansen T, Bennetzen M, Moller J, Pedersen SB, Richelsen B (2013). The macrophage-specific serum marker, soluble CD163, is increased in obesity and reduced after dietary-induced weight loss. Obesity (Silver Spring).

[28] Fjeldborg K, Pedersen SB, Moller HJ, Christiansen T, Bennetzen M, Richelsen B (2014). Human adipose tissue macrophages are enhanced but changed to an anti-inflammatory profile in obesity. J Immunol Res.

[29] Student AK, Hsu RY, Lane MD (1980). Induction of fatty acid synthetase synthesis in differentiating 3T3-L1 preadipocytes. J Biol Chem.

[30] Ntambi JM, Young-Cheul K (2000). Adipocyte differentiation and gene expression. J Nutr.

[31] Kajimoto K, Naraba H, Iwai N (2006). MicroRNA and 3T3-L1 pre-adipocyte differentiation. RNA.

[32] Gabrielsson BG, Olofsson LE, Sjogren A, Jernas M, Elander A, Lonn M (2005). Evaluation of reference genes for studies of gene expression in human adipose tissue. Obes Res.

[33] Vandesompele J, De PK, Pattyn F, Poppe B, Van RN, De PA (2002). Accurate normalization of real-time quantitative RT-PCR data by geometric averaging of multiple internal control genes. Genome Biol.

[34] Arnaud J, Touvier M, Galan P, Andriollo-Sanchez M, Ruffieux D, Roussel AM (2010). Determinants of serum zinc concentrations in a population of French middle-age subjects (SU.VI.MAX cohort). Eur J Clin Nutr.

[35] Sanchez C, Lopez-Jurado M, Planells E, Llopis J, Aranda P (2009). Assessment of iron and zinc intake and related biochemical parameters in an adult Mediterranean population from southern Spain: influence of lifestyle factors. J Nutr Biochem.

[36] Sayadi A, Nguyen AT, Bard FA, Bard-Chapeau EA (2013). Zip14 expression induced by lipopolysaccharides in macrophages attenuates inflammatory response. Inflamm Res.

[37] Nygaard SB, Larsen A, Knuhtsen A, Rungby J, Smidt K (2014). Effects of zinc supplementation and zinc chelation on in vitro beta-cell function in INS-1E cells. BMC Res Notes.

[38] Schmidt C, Beyersmann D (1999). Transient peaks in zinc and metallothionein levels during differentiation of 3T3L1 cells. Arch Biochem Biophys.

[39] Hogstrand C, Zheng D, Feeney G, Cunningham P, Kille P (2008). Zinc-controlled gene expression by metal-regulatory transcription factor 1 (MTF1) in a model vertebrate, the zebrafish. Biochem Soc Trans.

[40] Lichten LA, Ryu MS, Guo L, Embury J, Cousins RJ (2011). MTF-1-Mediated Repression of the Zinc Transporter Zip10 Is Alleviated by Zinc Restriction. PLoS One.

[41] Owen GI, Zelent A (2000). Origins and evolutionary diversification of the nuclear receptor superfamily. Cell Mol Life Sci.

[42] Kim TK, Park KS. Inhibitory effects of harpagoside on TNF-alpha-induced pro-inflammatory adipokine expression through PPAR-gamma activation in 3T3-L1 adipocytes. Cytokine. 2015; doi:10.1016/j.cyto.2015.05.015. 10.1016/j.cyto.2015.05.015 26049170

[43] Ye J (2008). Regulation of PPARgamma function by TNF-alpha. Biochem Biophys Res Commun.

[44] Trayhurn P (2005). Endocrine and signalling role of adipose tissue: new perspectives on fat. Acta Physiol Scand.

[45] Sewter C, Blows F, Considine R, Vidal-Puig A, O'Rahilly S (2002). Differential effects of adiposity on peroxisomal proliferator-activated receptor gamma1 and gamma2 messenger ribonucleic acid expression in human adipocytes. J Clin Endocrinol Metab.

[46] Fox CS, Massaro JM, Hoffmann U, Pou KM, Maurovich-Horvat P, Liu CY (2007). Abdominal visceral and subcutaneous adipose tissue compartments: association with metabolic risk factors in the Framingham Heart Study. Circulation.

[47] Liuzzi JP, Lichten LA, Rivera S, Blanchard RK, Aydemir TB, Knutson MD (2005). Interleukin-6 regulates the zinc transporter Zip14 in liver and contributes to the hypozincemia of the acute-phase response. Proc Natl Acad Sci U S A.

[48] Fruhbeck G (2008). Overview of adipose tissue and its role in obesity and metabolic disorders. Methods Mol Biol.

[49] Canatan H, Bakan I, Akbulut M, Halifeoglu I, Cikim G, Baydas G (2004). Relationship among levels of leptin and zinc, copper, and zinc/copper ratio in plasma of patients with essential hypertension and healthy normotensive subjects. Biol Trace Elem Res.

[50] Chen MD, Song YM, Lin PY (2000). Zinc may be a mediator of leptin production in humans. Life Sci.

[51] Konukoglu D, Turhan MS, Ercan M, Serin O (2004). Relationship between plasma leptin and zinc levels and the effect of insulin and oxidative stress on leptin levels in obese diabetic patients. J Nutr Biochem.

[52] Voruganti VS, Cai G, Klohe DM, Jordan KC, Lane MA, Freeland-Graves JH (2010). Short-term weight loss in overweight/obese low-income women improves plasma zinc and metabolic syndrome risk factors. J Trace Elem Med Biol.

[53] Ishikawa Y, Kudo H, Kagawa Y, Sakamoto S (2005). Increased plasma levels of zinc in obese adult females on a weight-loss program based on a hypocaloric balanced diet. In Vivo.

[54] Tungtrongchitr R, Pongpaew P, Phonrat B, Tungtrongchitr A, Viroonudomphol D, Vudhivai N (2003). Serum copper, zinc, ceruloplasmin and superoxide dismutase in Thai overweight and obese. J Med Assoc Thai.

[55] Ennes Dourado FF, de Sousa Lima VB, Mello Soares NR, Franciscato Cozzolino SM (2011). do Nascimento MD. Biomarkers of metabolic syndrome and its relationship with the zinc nutritional status in obese women. Nutr Hosp.

