# Supplementary material for: Gene expression of the zinc transporter ZIP14 (SLC39a14) is affected by weight loss and metabolic status and associates with PPARγ in human adipose tissue and 3T3-L1 pre-adipocytes
Source: BMC Obes. 2015 Nov 24;2:46. doi: 10.1186/s40608-015-0076-y (PMC4657294; doi:10.1186/s40608-015-0076-y)
Supplement: Additional file 1: — Primer sequences. Forward and reverse primer sequences are shown together with the annealing temperature. h, human primer; m, murine primer. In human adipose tissue, expression of the zinc transporter ZIP14 was investigated together with that of peroxisome proliferator-activated receptor γ isoform 1 (PPARγ1) and isoform 2 (PPARγ2). Low-density lipoprotein receptor-related protein 10 (LRP10) was used as a housekeeping gene. In 3T3-L1 cells, expression of the zinc transporter ZIP14 was investigated together with that of the adipocytic differentiation markers PPARγ and fatty-acid binding protein 4 (A-FABP). Cyclophilin A (Cyc-A), hypoxanthine guanine phosphoribosyl transferase (HPRT), and ubiquitin conjugase-7 (UBC-7) were used as housekeeping genes. (PDF 58 kb) [file 40608_2015_76_MOESM1_ESM.pdf]

Gene	Forward primer	Reverse primer	Annealing temperature (° C)
hZIP14	5´ GCT TAT GGA GAA CCA CCC CT 3´.	5´ AGG TTC CTG TGT CCT TGC AC 3´.	59
hPPARγ1	5` GTG GCC GCA GAT TTG AAA GAA 3´.	5´ CCA TTA CGG AGA GAT CCA CGG 3´.	58
hPPARγ2	5´ GCA AAC CCC TAT TCC ATG CT 3´.	5´ ACG GAG CTG ATC CCA AAG TT 3´.	57
hLRP10	5´ GCT CCC TGG ATG ACA CAG AG 3´.	5´ GAG AAG GAA CAG GCA GGC AG 3´	61
mZIP14	5´ TGG AAC CCT CTA CTC CAA CG 3´.	5´ CTG AGG GTT GAA GCC AAA AG 3´.	59
mPPARγ	5´ CAG CAT TTC TGC TCC ACA CTA TGA AG 3´.	5´ AGC AAG GCA CTT CTG AAA CCG 3´.	60
mA-FABP	5´ GGA TTT GGT CAC CAT CCG GTC 3´.	5´ CAT AAA CTC TTG TGG AAG TCA CGC C 3´.	62
mCyc-A	5´ GTG GTC TTT GGG AAG GTG AA 3´.	5´ TTA CAG GAC ATT GCG AGC AG 3´.	58
mHPRT	5´ AAG CTT GCT GGT GAA AAG GA 3´.	5´ TTG CGC TCA TCT TAG GCT TT 3´.	57
mUBC-7	5´ GGA ACT GGG CTG CAA TAA AA 3´.	5´ CCG GAT CAT GTT GTG CTA TG 3´.	58
