# Supplementary figures and images for: Gene expression of the zinc transporter ZIP14 (SLC39a14) is affected by weight loss and metabolic status and associates with PPARγ in human adipose tissue and 3T3-L1 pre-adipocytes
Source: BMC Obes. 2015 Nov 24;2:46. doi: 10.1186/s40608-015-0076-y (PMC4657294; doi:10.1186/s40608-015-0076-y)

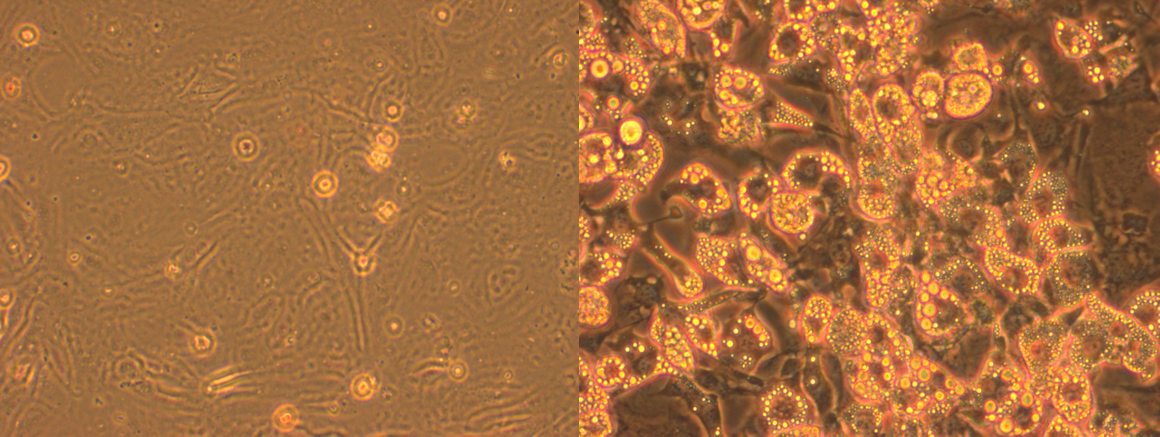

Supplement: Additional file 2: — Micrograph of undifferentiated (left side) and mature adipocytes (right side). Micrograph (×20) of 3T3-L1 cells prior to differentiation (left) and mature 3T3-L1 adipocytes 8 days after differentiation began (right). (TIF 1332 kb) [file 40608_2015_76_MOESM2_ESM.tif]
